# Barriers experienced by nurses providing smoking cessation support to disadvantaged, young women during and after pregnancy

**DOI:** 10.1111/hsc.12828

**Published:** 2019-08-25

**Authors:** Marloes E. Derksen, Anton E. Kunst, Monique W. M. Jaspers, Mirjam P. Fransen

**Affiliations:** ^1^ Department of Public Health Amsterdam UMC, University of Amsterdam, Amsterdam Public Health Research Institute Amsterdam Netherlands; ^2^ Department of Medical Informatics Amsterdam UMC, University of Amsterdam, Amsterdam Public Health Research Institute Amsterdam Netherlands

**Keywords:** deprivation, mothers, nurses, pregnancy, qualitative research, smoking cessation support

## Abstract

In Europe, smoking during and after pregnancy is still highly prevalent among socioeconomically disadvantaged women. Nurses caring for these women can play a key role in smoking cessation, but encounter many problems when providing support. This research aims to identify barriers in providing smoking cessation support, experienced by nurses working within a Dutch preventive care programme for disadvantaged young women (VoorZorg), and to understand the underlying reasons of these barriers. Sixteen semi‐structured interviews with nurses were performed. All interviews were recorded, transcribed and analysed deductively and inductively. We found that the VoorZorg programme provided nurses with training, resources and time to deliver smoking cessation support. Yet, nurses experienced important barriers, such as unmotivated clients and support methods that do not fit clients’ needs. Underlying reasons are competing care demands, unsatisfactory training for cessation support, lack of self‐efficacy in attending their clients, and conflicts with own professional attitudes. The results emphasise that nurses’ ability to provide smoking cessation support could be improved by proper training in interventions that fit their clients’ needs, and by time schedules and task definitions that help them to prioritise smoking cessation support over other matters.

**What is known about this topic**

- Various factors influence healthcare professionals’ provision of smoking cessation support (e.g. training, experience and time available).
- Healthcare professionals find it hard to provide smoking cessation support to socioeconomically disadvantaged women.

**What this paper adds**

- Barriers (e.g. clients’ lack of motivation to quit, nurses’ other priorities, inadequate training, support methods and lack of self‐efficacy) persist even within a preventive care programme specifically designed for attending to socioeconomically disadvantaged young women during and after pregnancy.
- The results emphasise the need to train nurses in smoking cessation support methods that fit clients’ needs and their poor motivation for smoking cessation. Also, the results underline that pregnancy care programmes should enable nurses to provide smoking cessation support next to the many other priorities.

## 1 INTRODUCTION

About a quarter of European women that smoke before their pregnancy, continue smoking during their pregnancy (Smedberg, Lupattelli, Mårdby, & Nordeng, 2014). Maternal cigarette smoking during pregnancy is associated with orofacial clefts, still birth, low birth weight, preterm birth and sudden infant death syndrome (U.S. Department of Health & Human Services, 2014), and a wide range of health consequences later in life (e.g. asthma, wheeze, and weight related health problems; Bell et al., 2018). Children's exposure to second‐hand smoke after birth is causally linked to sudden infant death syndrome, middle ear disease and respiratory diseases (U.S. Department of Health and Human Services, 2014).

The transtheoretical model explains that health behaviour change (i.e. smoking cessation) involves moving through stages of change ranging from precontemplation to termination (Prochaska & Velicer, 1997). Professionals caring for pregnant women can play a key role in helping women move through the stages of change by providing smoking cessation support, as pregnancy is found to be a naturally occurring teachable moment for smoking cessation (McBride, Emmons, & Lipkus, 2003). Moreover, healthcare professionals can include the post‐partum period in smoking cessation support, as women may want to quit smoking after giving birth in order to protect their baby from second‐hand smoke. Furthermore, many women relapse in this period (DiClemente, Dolan‐Mullen, & Windsor, 2000).

Various factors influence healthcare professionals’ provision of smoking cessation support such as training, experience, task perception and time available (Flemming et al., 2016; Svavarsdóttir & Hallgrímsdóttir, 2008). Healthcare professionals find it hard to provide smoking cessation support to socioeconomically disadvantaged women, as many of these women have strongly embedded smoking habits, and perceive smoking as a relief from daily stressors. Moreover, healthcare professionals feel that they cannot address the social problems these women face. As a result, professionals expect that the majority of women would not succeed in quit attempts (Flemming et al., 2016).

Given the above, it is important to understand how healthcare professionals can best be supported in the provision of smoking cessation support to socioeconomically disadvantaged women (Smedberg et al., 2014). Evidence on factors influencing healthcare providers’ provision of smoking cessation support is limited and mostly derived from studies among expectant women instead of healthcare professionals themselves (Flemming, McCaughan, Angus, & Graham, 2015; Schneider, Huy, Schuetz, & Diehl, 2010). A study that was performed among nurse‐midwives working in clinical settings found that most nurses did not report barriers in providing smoking cessation support (Price, Jordan, & Dake, 2006).

In a Dutch preventive care programme, VoorZorg, nurses provide smoking cessation support to disadvantaged young women during and after pregnancy. VoorZorg has similarities to the Nurse–Family partnership developed in the United States (Olds, 2006) and the Family–Nurse Partnership implemented in the United Kingdom (Robling et al., 2016). The programme targets socioeconomically disadvantaged women, that at the time of registration are (a) at 28 or less weeks of gestation (with no previous live births), (b) no older than 25 years of age, (c) lower level of education, (d) proficient in the Dutch language and (e) face at least one additional risk factor (e.g. lack of social network, financial problems, psychosocial symptoms, drug/alcohol use and domestic violence). Certified specialised nurses support these women during home visits in a period of 2.5 years on multiple domains, such as health promotion and personal development (Mejdoubi et al., 2014). VoorZorg is offered in addition to standard maternal care in the Netherlands and includes regular check‐ups by midwives or obstetricians (Mejdoubi et al., 2013). Clients may also receive help/support from other organisations, including youth care and mental healthcare, on issues such as finances, social work, domestic violence and child abuse.

Currently, the provision of smoking cessation support is standard unconditional care within the full VoorZorg programme. It includes inquiring about clients' smoking behaviour and exposure to passive cigarette smoking during every home visit. Nurses are instructed and trained to offer an intervention, named “V‐MIS” to clients that smoke. Duration of the intervention depends on the pace with which the client proceeds through V‐MIS. V‐MIS, also used by Dutch midwives and gynaecologists, is a minimal intervention strategy based on the Integrated Change Model and transtheoretical model, adapted to pregnant women (de Vries, Bakker, Mullen, & van Breukelen, 2006; see Box; Bakker, Mullen, de Vries, & van Breukelen, 2003).

Box 1 **Seven steps of V‐MIS (Bakker et al., 2003)**

1 Step 1. Classifying pregnant women to their stage of change, the first step is to identify her (and her partners’) smoke profile and motivation to quit smoking.

Step 2. Increasing the motivation of the pregnant woman to quit smoking, adjusted to women's stage of change.

Step 3. Discussing and removing barriers to smoking cessation and mobilising support in the immediate environment (directed at women in the preparation stage).

Step 4. Choosing a date with the pregnant woman (and her partner) for smoking cessation (preparation stage).

Step 5. Distributing a brochure and flyer of tailored advice to pregnant woman and discussing potential useful tools (executed to women at all stages of change).

Step 6. Following consultations after quit date, returning to the topic smoking cessation and providing after care (action stage).

Step 7. Preventing of relapse after delivery (executed to women at all stages of change).

A previous study showed that at the onset of the VoorZorg programme (16–28 weeks of gestation), 43% of the clients smoked cigarettes. This was reduced to 33% at 32 weeks of gestation. Two months after birth, 48% of VoorZorg clients smoked cigarettes (Mejdoubi et al., 2014). Thus, although the prevalence of clients that smoked during pregnancy reduced, this reduction was modest and reverted after birth.

Our aims were to assess the barriers that nurses experience in providing smoking cessation support to socioeconomically disadvantaged young women during and after pregnancy, and to understand the underlying reasons of these barriers.

## 2 METHODS

In this observational study, we conducted semi‐structured qualitative interviews among nurses who provided smoking cessation support to disadvantaged young women during and after pregnancy within the VoorZorg context. All participants gave their informed consent verbally, which was audio‐recorded. The Medical Ethics Review Committee of Amsterdam UMC confirmed that the Medical Research Involving Human Subjects Act does not apply to this study, and thus no official approval of the committee was required.

### 2.1 Participants and recruitment

The study population consisted of nurses. Inclusion criteria were that nurses should be employed as VoorZorg nurse in the Netherlands, and have at least 6 months experience in providing smoking cessation support to VoorZorg clients. Managers of Youth Health Care organisations executing the VoorZorg programme were informed about the study and asked for permission of their nurses to participate in this study. Permission was granted by nine managers. Four managers denied permission due to time constraints (*N* = 2), and due to insufficient experience (*N* = 2). Subsequently, nurses were informed about the study during a conference and were later asked by email whether they wanted to participate in the study. A reminder email was sent to nurses that had not replied. Inclusion was based on consecutive sampling.

Sixteen out of 36 nurses meeting the inclusion criteria participated (participation rate 44%). They worked at eight different organisations scattered over six regions in the Netherlands. Most non‐participants did not respond to the email invitation, the four non‐participants that did respond indicated that they denied participation due to other priorities (*N* = 2) or due to limited experience in providing smoking cessation support (*N* = 2).

### 2.2 Data collection

Data were collected by telephone interviews (conducted by MD) held between June and October 2017. One interview was face‐to‐face and took place at the workplace of the nurse. All interviews were in Dutch. Interviews were one‐off and lasted approximately 45 min. Since data were collected during work hours, organisations received a compensation of €25, per participating nurse.

Participants were informed that the research aimed to gain insight into smoking cessation support that participation was voluntary, and that data would be anonymised. Interviews were audio recorded and field notes were made. A semi‐structured interview guide was used, based on the framework of Fleuren, Wiefferink, and Paulussen (2002) which describes that, applied to our research, provision of smoking cessation support (i.e. implementation) can be influenced by characteristics of (a) the context of VoorZorg, (b) providing smoking cessation support, and (c) nurses. Topics covered in the interview guide were the current practice of smoking cessation support and perceived facilitators and barriers of the current practice based on their full work experience. Afterwards, participants completed a short questionnaire to collect baseline characteristics.

### 2.3 Data analysis

Raw interview data were transcribed verbatim, except the data of one participant due to a failed recording. Prior to coding the data, a coding tree was prepared based on the interview guide (i.e. framework developed by Fleuren et al. (2002), see above), literature(Flemming et al., 2016; Seeleman, Suurmond, & Stronks, 2009) and our research questions (see Appendix I). MD analysed all data in three rounds of coding, using MAXQDA 12. Data analysis was an iterative process. First, data were coded deductively using the predefined coding tree (i.e. directed content analysis; Hsieh & Shannon, 2005). Data analysis was complemented with an inductive approach: by constant comparison within and between interview transcripts. Newly emerging codes were added to the coding tree (i.e. open coding; Boeije, 2009) and applied to pieces of text that could not be coded with codes from the initial coding tree. Previously coded interviews were reanalysed for presence of the newly added codes. Next, relationships between codes were (re)defined: generating core‐ and subthemes (i.e. axial and selective coding; Boeije, 2009).

MF randomly and independently coded three interview transcripts in the first round of coding. MF and MD agreed on the majority of the coding, and the remaining disagreements between authors were resolved in a consensus session. These disagreements entailed pieces of text coded by one author, but not the other and vice versa. Regular research meetings were held to foster reflexivity in the coding process. Authors agreed that saturation was reached, at the moment that no new themes emerged during the last two interviews. After data analysis, a native English speaker translated citations from the transcripts to English.

## 3 FINDINGS

### 3.1 Participant characteristics

Nurses in our sample were on average 47 years old (range 31–62) and had 7 years of experience as VoorZorg nurse (range 0.5–11). Nurses worked an average of 20 hr per week as VoorZorg nurse. At the time of this study, nurses took care of 143 clients in total, of which 51 (35.7%) were current smokers.

### 3.2 Themes

Data analysis resulted in three themes of nurses’ perceptions and experiences in providing smoking cessation support to socioeconomically disadvantaged young women during and after pregnancy: (a) the VoorZorg context, (b) barriers in providing smoking cessation support, and (c) underlying reasons.

#### 3.2.1 Theme 1 ‐ The VoorZorg context

Nurses considered providing smoking cessation support their responsibility. While the planned home visits are typically rich in topics that need to be discussed, nurses experienced no time constrains to provide smoking cessation support:

I have to do it [provide smoking cessation support], it is my responsibility to do it [provide smoking cessation support]. (Participant 13)

Because there is a lot of time and opportunities [in VoorZorg programme], there are many possibilities to discuss a variety of topics. Something that can’t be accomplished today, can be accomplished next time. (Participant 3)

Furthermore, the frequent and continued moments of contact during the period of care, allowed nurses and clients to build a trusting relationship. Nurses viewed this as constructive since sensitive topics such as smoking are discussed more easily:

I think what works well is that we build a good relationship with each other, and that from there you can ask more and maybe you can say that it works better [to provide smoking cessation support]. (Participant 6)

In addition, nurses mentioned being equipped with and supported by materials of the VoorZorg programme to provide smoking cessation support and being trained to use these materials. Lastly, nurses emphasised that the way of providing smoking cessation support (i.e. motivational interviewing, empowering techniques) is used throughout the VoorZorg programme, making it compatible with the overall programme.

#### 3.2.2 Theme 2 – Barriers in providing smoking cessation support

Nurses immediately came across barriers following the implementation of V‐MIS (i.e. step 1 identifying the smoke profile/stage of change of a client). Since client support starts early after enrolment in the programme, nurses explained that they could not rely on their trusting relationship with clients in discussing sensitive topics such as smoking cessation, at that point. Clients are generally reluctant to discuss their smoking habits at that stage:

They really don’t want you to come with a thing about smoking. (Participant 3)

Nurses also noticed that they tended to have the least motivated smoking clients with high levels of addiction, because more motivated clients already quit smoking before the onset of the VoorZorg programme:

If they [clients] are [motivated to quit smoking], than they have often quit before we come into the picture, because they are able to flip a switch themselves and then we [nurses] do not have to do anything. It seems like either they quit, or they cannot quit and it will not happen. (Participant 5)

With this unmotivated group of smoking clients, nurses found it very hard to enter the next steps of V‐MIS, as they perceived that their clients should at least be somewhat motivated to continue providing smoking cessation support.

Most nurses indicated that they get stuck in step 2 of V‐MIS, which entails increasing clients’ intrinsic motivation to quit smoking, adjusted to clients’ stage of change. Nurses believed that clients have other urgent matters on their minds and smoking often serves as a coping mechanism to deal with those matters:

The motivation of the girls to quit is just not there. I try to empower them and to look at possible advantages together and things like that. But if they really don’t see it, I get stuck. (Participant 8)

That intrinsic motivation is very difficult to initiate in clients. There are often so many miserable things going on in their lives that that [smoking] is their only safeguard. (Participant 14)

Because they [clients] are often so busy with the child, the pregnancy, actually with surviving. I think that is a cause that it is very difficult to find the motivation to fully quit. (Participant 10)

After attempting to increase clients’ motivation, V‐MIS prescribes to discuss and ideally resolve clients’ barriers for quitting smoking (according to V‐MIS step 3, preparation stage). Nurses explained that clients express many arguments that prevent them from making quit attempts (e.g. “My mother smoked and I am healthy.”). When trying to resolve these barriers with clients, nurses felt stranded in these discussions, and often did not succeed in getting their message across:

I notice that I, how should I say that, get stuck on discussing smoking because I keep running into all of the same arguments. (Participant 14)

In step 3, nurses and clients are also supposed to make a plan of how to overcome the clients’ barriers. This is in itself an obstacle to clients, nurses explained that the support becomes theoretical rather than practical, resulting in a loss of interest among the clients:

They [clients] know very well when their need is stronger and when/where their pitfalls lie. They realise that. But I think it is more: if you make a plan then it becomes a bit theoretical and most of the time they lose interest at that point. (Participant 3)

But it is also a barrier to nurses: nurses described once again that many things happen in the lives of the clients, causing plans to fail. This results in disappointment among clients, which nurses wanted to protect them from:

When making a plan, you have to think about when it might go wrong. But often so many things already go wrong in their lives, that the plans often already fail. And that sounds incredibly negative, but that is often the reality. Whereby it [smoking cessation] is often such a disappointment, and that is what you want to protect the girls from. (…) That is a barrier: at a certain point you have to go through the various steps, but 9 out of 10 times, it is a disappointment. (Participant 3)

The last task in step 3 is to mobilise social support in clients’ immediate environment. According to nurses, this mobilisation is impeded since most of the relatives and friends in the direct social environment of clients smoke themselves. This also makes it harder for clients to achieve (prolonged) smoking cessation, and for nurses to provide support:

When I come over and discuss it [smoking cessation], the whole family is smoking in the living room. And they really do not care when I tell them that smoking is not healthy. Because in their family all children were born healthy, thus it must be nonsense. That of course makes me feel a bit discouraged. (Participant 16)

Selecting a date on which clients will quit smoking (according to V‐MIS step 4, preparation stage) was considered to be challenging as well. Nurses explained that clients tend to postpone their quit date, because the discussion of potential barriers in the previous step causes feelings of stress:

Sometimes when you discuss it in advance [barriers for smoking cessation], then they will already get stressed out, and thereby postpone setting a quit date again. (Participant 10)

When nurses and clients do select a quit date, clients are likely to face a drawback in their lives. This often leads to a failed quit attempt and disappointed clients that give up:

They choose a [quit] date, but of course something will always happen, whereby it doesn’t work out and then they are often so disappointed. Because then they say: ‘See, I can’t do it’. And then they just give up. (Participant 10)

Nurses mentioned that they generally discuss and/or provide clients with additional support for smoking cessation (e.g. brochures, referral to smoking cessation specialist; according to V‐MIS step 5, clients in all stages of change). Although V‐MIS does provide materials, these seem insufficient. Some nurses said that they use other flyers and images, or looked up videos on the internet themselves in an attempt to get their message across:

I just showed videos on YouTube. (…) But, that is not really what is offered within VoorZorg, that really is what I looked up on the internet myself. (Participant 3)

Moreover, nurses expressed that they do not feel sufficiently knowledgeable to provide clients with additional information (e.g. on nicotine replacement therapy):

Tools, temporary solutions, or nicotine chewing gum and such, I would like to know more about that. So that I could help them better with that. (Participant 10)

One nurse stated that visiting a smoking cessation specialist does not fit her clients’ needs (clients have other priorities than doing that). She therefore does not refer her clients:

I find the referring to a specialist, our clients can’t handle that. They will not do that. (…) Our clients do not take that step, they have so many bigger concerns than that. (Participant 12)

After completing the previous steps, nurses have to monitor the smoking cessation process (according to V‐MIS step 6, action stage) and work on relapse prevention (according to V‐MIS step 7, all stages of change). No barriers were mentioned by nurses in these steps of V‐MIS.

#### 3.2.3 Theme 3 – underlying reasons

##### Contextual barriers

A frequently reported underlying reason for barriers to provide smoking cessation support within VoorZorg, was that other (more urgent) issues are prioritised. Clients often wanted to discuss other topics that are more important to them than smoking. It seems that nurses want to comply with these needs, but also that nurses themselves are convinced that other matters often have more priority than providing smoking cessation support:

I often feel that there are so many other things that also require attention [besides providing smoking cessation support]. And [things] that the girls want to talk about (…) and that are actually a much greater risk to the child or to the mother. (Participant 6)

Another underlying reason was that nurses found their V‐MIS training unfulfilling. The training was rather short and while the steps seemed clear, it turned out to be hard to apply in practice, as the training was not adapted to their context:

At that moment I found it [training in providing smoking cessation support by using V‐MIS] very clear, but I found it difficult to apply it in practice. (Participant 8)

Furthermore, nurses shed light on the intricacies of their relationship with clients. As explained above, the relationship with their clients could help smoking cessation support, but could also seriously harm the relationship as it often is such a sensitive topic:

I have to really sense when can I, when can’t I, how often can I, how often can’t I [provide smoking cessation support]… It could destroy your relationship if you go too far, or do not handle it the right way. (Participant 14)

They stressed that the way they provide support (e.g. their tone) is of great importance as it could easily provoke resistance among their clients:

That is the way you make it [smoking cessation] discussable, that is important. That is what clients tell us, for example the midwife tells them (…) “you have to quit smoking because it is bad”. So that way of saying that to them (…) provokes resistance. (Participant 1)

Nurses also experienced situations in which clients claimed that their midwife/gynaecologists permitted them to smoke a few cigarettes:

She even says that the midwives and gynaecologist told her to smoke a few cigarettes otherwise she would get too stressed out. (Participant 13)

Yet, nurses are convinced that other healthcare professionals would not permit smoking during pregnancy:

That is not true, I know that for sure. Because every midwife says that you’re not allowed to smoking during pregnancy. (Participant 4)

Nevertheless, nurses seemed to realise that whether other healthcare professionals actually do or do not tell clients this, they have to refute this perception and that it is their word against that of a professional regarded as more of an expert in pregnancy matters.

##### Barriers in provision of smoking cessation support

Although not mentioned as a barrier by nurses themselves, generally nurses were aware of the content and execution of V‐MIS, but unaware of the specific steps of V‐MIS:

I would not even know exactly what the different steps are, I know the basics, the idea. (Participant 6)

Nevertheless, nurses noticed that V‐MIS does not fit with the clients’ needs and circumstances and the VoorZorg programme. The plan‐based approach does not allow for other aspects in clients’ lives to be addressed:

It [V‐MIS] is has quite strict steps. That actually does not fit well with VoorZorg, in my opinion. (Participant 12)

It could be the case that you started such a plan [V‐MIS], and then there are many fires to put out. Subsequently the plan has not been touched for two months and you have to start over again. (Participant 5)

Consequently, nurses predominantly developed their own methods for providing smoking cessation support:

We work with V‐MIS. But I notice that it does not fit with our clients and therefore I often leave it for what it is. And that I just start a conversation. And that it comes more from me, rather than I really use the materials that are provided [i.e. V‐MIS]. (Participant 3)

##### Barriers in personal determinants of nurses

Nurses’ lacked (complete) self‐efficacy in providing smoking cessation support:

I do not feel competent enough to do that [providing smoking cessation support]. (Participant 6)

This is reflected by the difficulties that nurses experience having to adjust their cessation support to the low health literacy skills of their clients:

My inability to transfer, or rather the inability of parents to understand what I explain about the risks for their children by their smoking behaviour. (Participant 14)

It also seemed that nurses’ attitude towards providing smoking cessation support has changed due to their experiences in VoorZorg. While nurses initially aimed to provide smoking cessation support, they felt their provision of smoking cessation support was overly persistent. This resulted in aiming for smaller achievements (i.e. not smoking indoors):

Sometimes I find it a bit ‘whining’ myself. Then I think: they already know it and yet I come by with another talk on smoking [cessation]. (Participant 13)

My standards have changed a bit. So I do not have mothers that smoke in the presence of their child, well I find that really pleasant. (Participant 6)

## 4 DISCUSSION

This qualitative study among nurses showed that nurses experience important barriers in their roles, such as competing care demands, unsatisfactory training for cessation support, support methods that do not fit to clients’ needs, lack of self‐efficacy in attending their clients and conflicts with own professional attitudes. They also felt that clients were poorly motivated to stop smoking, due to multiple stressors and other challenges they had to face.

### 4.1 Interpretation of findings

The VoorZorg programme provided a promising context for smoking cessation support to nurses by offering training, resources and time to deliver smoking cessation support. Barriers were found in the V‐MIS intervention available to nurses, which did not meet clients’ needs for smoking cessation support. Due to this lack of congruence, most nurses experience difficulties in implementing V‐MIS. We found that most barriers were face in the first steps of V‐MIS. This is in accordance to the findings of other studies that found that, when moving through the stages of V‐MIS, more and more healthcare professionals did not make it to the next step(Segaar, Willemsen, Bolman, & De Vries, 2007; Oude Wesselink, Lingsma, Robben, & Mackenbach, 2015). In terms of the transtheoretical model of health behaviour change, clients of nurses in our study were at the (pre)contemplation stage, that is, not ready or unmotivated to change their smoking behaviour), as was observed in another study (Bull, 2007). V‐MIS, appears to undervalue addressing the early stages, like most traditional health promotion programmes (Prochaska & Velicer, 1997). V‐MIS only attends these stages in step 2, when healthcare professionals attempt to increase clients motivation, following steps of V‐MIS are directed to preparation and action stages.

Nurses reported that relatives and friends of clients are a barrier early in the process of provision of smoking cessation support as they often smoke themselves. Other qualitative research showed that having a partner, close family or friends that smoke could impede smoking cessation of women that smoked during pregnancy as they provide temptation to the women (Koshy, Mackenzie, Tappin, & Bauld, 2010). The importance of social context is also underlined in the Theory of Planned Behaviour which states that subjective norms of relevant others, besides attitudes and perceived behavioural control, influences behavioural intention and consequent behaviour (Ajzen, 1991). We also found that nurses often prioritised other matters over smoking cessation. This finding seems in contrast to the finding of Flemming et al. (2016), who found that healthcare professionals provide smoking advice to their clients even without support of the latter, because they felt this was their professional obligation. This may differ from our study in that our nurses were concerned with the lives of their clients in general, and all problems they have to cope with (e.g. housing and financial problems, lack of social network, psychosocial symptoms, substance use and domestic violence). Rather than being exclusively concerned with matters directly linked to pregnancy and physical health. Moreover, nurses reported inadequate training in smoking cessation support. Training of healthcare professionals in providing smoking cessation has been found to increase their readiness to carry out tasks, such as discussing quit dates and providing smoking cessation materials (Carson et al., 2012). We also found that, as the relationship between nurses and their clients is delicate, an emphatic provision of smoking cessation support might risk affecting their relationship. Flemming et al. (2016), who found that the lack of such a relationship constitutes a barrier to providing cessation support, underlines the importance of a positive, supportive relationship. Lastly, a concern mentioned by nurses in our study was that their clients pleaded that other healthcare professionals allowed them to continue to smoke a few cigarettes. A systematic review on pregnant women's views also found that women claimed that their healthcare professional permitted cutting down as an option besides to cessation (Graham, Flemming, Fox, Heirs, & Sowden, 2014). However, it remains uncertain whether this practice is widespread in The Netherlands. The Dutch National Expert Centre Tobacco Control disseminates to professionals the message that they should support their clients in complete smoking abstinence, no recommendations should be made on lowering tobacco consumption (Nationaal Expertisecentrum Tabaksontmoediging, 2017).

### 4.2 Limitations

This study was subject to some limitations. The unique context of VoorZorg can make it hard to apply our findings to other contexts. It is likely that, without the support that nurses received from the VoorZorg programme, nurses would report additional barriers such as time constrains and lack of organisational policies and resources (Flemming et al., 2016). Another limitation inherent to qualitative research is some degree of subjectivity in the analysis and interpretation of the data. We aimed to decrease interrater subjectivity by involving a second coder in the data analysis and by regular research meetings with all authors to discuss interim results.

### 4.3 Implications for practice

Our results highlight the need for smoking cessation interventions in which clients are helped to proceed through the early stages of change, that is, intending to change their smoking behaviour in the immediate future. Several methods can be used to help clients proceed through these early stages, including consciousness raising (e.g. feedback), dramatic relief (e.g. personal testimonies), environmental re‐evaluation (e.g. empathy training) and self‐re‐evaluation (e.g. healthy role models; Shumaker, Ockene, & Riekert, 2008). A method to apply these approaches in the context of care for women during and after pregnancy could be digital interventions, which have a potential to improve smoking cessation rates in pregnancy (Griffiths et al., 2018). Moreover, the results emphasise that interventions should have a holistic approach that allow women to achieve readiness for change, that is, resolving other priorities and increasing self‐efficacy for smoking cessation. In addition, our results highlight the importance of adequate training for healthcare professionals in providing smoking cessation support to disadvantaged women. Moreover, the organisational context should aid healthcare professionals in prioritising provision of smoking cessation support, e.g. by making smoking cessation support an explicit organisational goal, and by allowing for tailor‐made approaches to diverse clients (Flemming et al., 2016).

### 4.4 Conclusions

Our study revealed that even though the VoorZorg programme facilitated nurses with smoking cessation support, they faced persistent barriers in providing this support to disadvantaged young women during and after pregnancy. Nurses both experienced barriers during provision of smoking cessation support but also underlying reasons impeded provision of smoking cessation support. Nurses’ ability to provide smoking cessation support could be improved by adequate training of healthcare professionals, and the implementation of interventions that fit their clients’ needs. These interventions will especially need to focus on improving determinants of early stages of behavioural change.

## CONFLICT OF INTEREST

No conflicts of interest have been declared.

## APPENDIX I Predefined coding tree

### I.1 CATEGORY 1: CHARACTERISTICS OF ORGANIZATION OF CARE

Material resources and facilities

Education

Time

Formal ratification by management

### I.2 CATEGORY 2: CHARACTERISTICS OF PROVIDING SMOKING CESSATION SUPPORT

Compatibility

Complexity

Relevance for client

Procedural clarity

Correctness

Completeness

Observability

### I.3 CATEGORY 3: CHARACTERISTICS OF NURSES

Attitude

Personal benefits/drawbacks

Outcome expectations & realizations

Skills

Awareness of content of SPCS

Knowledge

Client satisfaction & cooperation

Social support

Social norms

Self‐efficacy

## References

[hsc12828-bib-0001] Ajzen, I. (1991). The theory of planned behavior. Organizational Behavior and Human Decision Processes, 50(2), 179–211. 10.1016/0749-5978(91)90020-T

[hsc12828-bib-0002] Bakker, M. J. , Mullen, P. D. , de Vries, H. , & van Breukelen, G. (2003). Feasibility of implementation of a Dutch smoking cessation and relapse prevention protocol for pregnant women. Patient Education and Counseling, 49(1), 35–43. 10.1016/S0738-3991(02)00038-1 12527151

[hsc12828-bib-0003] Bell, K. , Corbacho, B. , Ronaldson, S. , Richardson, G. , Torgerson, D. , & Robling, M. (2018). The impact of pre and perinatal lifestyle factors on child long term health and social outcomes: A systematic review. Health Economics Review, 8(1), 2 10.1186/s13561-018-0186-6 29368151 PMC5783983

[hsc12828-bib-0004] Boeije, H. (2009). Analysis in qualitative research. London, UK: Sage Publications.

[hsc12828-bib-0005] Bull, L. (2007). Smoking cessation intervention with pregnant women and new parents (part 2): A focus group study of health visitors and midwives working in the UK. Journal of Neonatal Nursing, 13(5), 179–185. 10.1016/j.jnn.2007.07.003

[hsc12828-bib-0006] Carson, K. V. , Verbiest, M. E. , Crone, M. R. , Brinn, M. P. , Esterman, A. J. , Assendelft, W. J. , & Smith, B. J. (2012). Training health professionals in smoking cessation. Cochrane Database of Systematic Reviews(5), 10.1002/14651858.CD000214.pub2 PMC10088066 22592671

[hsc12828-bib-0007] de Vries, H. , Bakker, M. , Mullen, P. D. , & van Breukelen, G. (2006). The effects of smoking cessation counseling by midwives on Dutch pregnant women and their partners. Patient Education and Counseling, 63(1–2), 177–187. 10.1016/j.pec.2005.10.002 16406475

[hsc12828-bib-0008] DiClemente, C. C. , Dolan‐Mullen, P. , & Windsor, R. A. (2000). The process of pregnancy smoking cessation: Implications for interventions. Tobacco Control, 9(Suppl. 3), 16iii–21. 10.1136/tc.9.suppl_3.iii16 10982900 PMC1766302

[hsc12828-bib-0009] Flemming, K. , Graham, H. , McCaughan, D. , Angus, K. , Sinclair, L. , & Bauld, L. (2016). Health professionals' perceptions of the barriers and facilitators to providing smoking cessation advice to women in pregnancy and during the post‐partum period: A systematic review of qualitative research. BMC Public Health, 16, 290 10.1186/s12889-016-2961-9 27030251 PMC4815177

[hsc12828-bib-0010] Flemming, K. , McCaughan, D. , Angus, K. , & Graham, H. (2015). Qualitative systematic review: Barriers and facilitators to smoking cessation experienced by women in pregnancy and following childbirth. Journal of Advanced Nursing, 71(6), 1210–1226. 10.1111/jan.12580 25430626

[hsc12828-bib-0011] Fleuren, M. A. H. , Wiefferink, C. H. , & Paulussen, T. G. W. M. (2002). Belemmerende en bevorderende factoren bij de implementatie van zorgvernieuwingen in organisaties. Leiden, the Netherlands: TNO Preventie en Gezondheid, Divisie Volksgezondheid.

[hsc12828-bib-0012] Graham, H. , Flemming, K. , Fox, D. , Heirs, M. , & Sowden, A. (2014). Cutting down: Insights from qualitative studies of smoking in pregnancy. Health & Social Care in the Community, 22(3), 259–267. 10.1111/hsc.12080 24224830

[hsc12828-bib-0013] Griffiths, S. E. , Parsons, J. , Fulton, E. A. , Naughton, F. , Tombor, I. , & Brown, K. E. (2018). Are digital interventions for smoking cessation in pregnancy effective? A systematic review and meta‐analysis. Health Psychology Review, 1–52, 10.1080/17437199.2018.1488602 29912621

[hsc12828-bib-0014] Hsieh, H. F. , & Shannon, S. E. (2005). Three approaches to qualitative content analysis. Qualitative Health Research, 15(9), 1277–1288. 10.1177/1049732305276687 16204405

[hsc12828-bib-0015] Koshy, P. , Mackenzie, M. , Tappin, D. , & Bauld, L. (2010). Smoking cessation during pregnancy: The influence of partners, family and friends on quitters and non‐quitters. Health & Social Care in the Community, 18(5), 500–510. 10.1111/j.1365-2524.2010.00926.x 20561076

[hsc12828-bib-0016] McBride, C. M. , Emmons, K. M. , & Lipkus, I. M. (2003). Understanding the potential of teachable moments: The case of smoking cessation. Health Education Research, 18(2), 156–170. 10.1093/her/18.2.156 12729175

[hsc12828-bib-0017] Mejdoubi, J. , van den Heijkant, S. , Struijf, E. , van Leerdam, F. , HiraSing, R. , & Crijnen, A. J. (2013). Risicofactoren voor kindermishandeling bij jonge hoogrisicozwangeren: Design van het VoorZorgonderzoek. JGZ Tijdschrift Voor Jeugdgezondheidszorg, 45(2), 26–31. 10.1007/s12452-013-0007-6

[hsc12828-bib-0018] Mejdoubi, J. , van den Heijkant, S. , van Leerdam, F. J. M. , Crone, M. , Crijnen, A. , & HiraSing, R. A. (2014). Effects of nurse home visitation on cigarette smoking, pregnancy outcomes and breastfeeding: A randomized controlled trial. Midwifery, 30(6), 688–695. 10.1016/j.midw.2013.08.006 24041564

[hsc12828-bib-0019] Nationaal Expertisecentrum Tabaksontmoediging . (2017). Factsheet Minderen met roken. Retrieved from https://assets.trimbos.nl/docs/94dce4af-b08b-49f0-9afe-301042aed4af.pdf

[hsc12828-bib-0020] Olds, D. L. (2006). The nurse‐family partnership: An evidence‐based preventive intervention. Infant Mental Health Journal, 27(1), 5–25. 10.1002/imhj.20077 28640426

[hsc12828-bib-0021] Oude Wesselink, S. F. , Lingsma, H. F. , Robben, P. B. M. , & Mackenbach, J. P. (2015). Provision and effect of quit-smoking counselling by primary care midwives. Midwifery, 31(10), 986–992. 10.1016/j.midw.2015.05.010. 26119832

[hsc12828-bib-0022] Price, J. H. , Jordan, T. R. , & Dake, J. A. (2006). Perceptions and use of smoking cessation in nurse‐midwives’ practice. Journal of Midwifery & Women's Health, 51(3), 208–215. 10.1016/j.jmwh.2005.12.003 16647673

[hsc12828-bib-0023] Prochaska, J. O. , & Velicer, W. F. (1997). The transtheoretical model of health behavior change. American Journal of Health Promotion, 12(1), 38–48. 10.4278/0890-1171-12.1.38 10170434

[hsc12828-bib-0024] Robling, M. , Bekkers, M.‐J. , Bell, K. , Butler, C. C. , Cannings‐John, R. , Channon, S. , … Torgerson, D. (2016). Effectiveness of a nurse‐led intensive home‐visitation programme for first‐time teenage mothers (Building Blocks): A pragmatic randomised controlled trial. Lancet, 38(10014), 146–155. 10.1016/S0140-6736(15)00392-X PMC4707160 26474809

[hsc12828-bib-0025] Schneider, S. , Huy, C. , Schuetz, J. , & Diehl, K. (2010). Smoking cessation during pregnancy: A systematic literature review. Drug and Alcohol Review, 29(1), 81–90. 10.1111/j.1465-3362.2009.00098.x 20078687

[hsc12828-bib-0026] Seeleman, C. , Suurmond, J. , & Stronks, K. (2009). Cultural competence: A conceptual framework for teaching and learning. Medical Education, 43(3), 229–237. 10.1111/j.1365-2923.2008.03269.x 19250349

[hsc12828-bib-0027] Segaar, D. , Willemsen, M. C. , Bolman, C. , & De Vries, H. (2007). Nurse adherence to a minimal‐contact smoking cessation intervention on cardiac wards. Research in Nursing & Health, 30(4), 429–444. 10.1002/nur.20204 17654478

[hsc12828-bib-0028] Shumaker, S. A. , Ockene, J. K. , & Riekert, K. A. (2008). The handbook of health behavior change (3rd ed.). New York, NY: Springer Publishing Company.

[hsc12828-bib-0029] Smedberg, J. , Lupattelli, A. , Mårdby, A. , & Nordeng, H. (2014). Characteristics of women who continue smoking during pregnancy: A cross‐sectional study of pregnant women and new mothers in 15 European countries. BMC Pregnancy and Childbirth, 14(1), 213 10.1186/1471-2393-14-213 24964728 PMC4080751

[hsc12828-bib-0030] Svavarsdóttir, M. H. , & Hallgrímsdóttir, G. (2008). Participation of Icelandic nurses in smoking cessation counselling. Journal of Clincal Nursing, 17(10), 1335–1341. 10.1111/j.1365-2702.2006.01874.x 17419789

[hsc12828-bib-0031] U.S. Department of Health and Human Services . (2014). The health consequences of smoking—50 years of progress: A report of the Surgeon General. Atlanta, GA: US Department of Health and Human Services. Centers for Disease Control and Prevention, National Center for Chronic Disease Prevention and Health Promotion, Office on Smoking and Health.

